# Stricture of the Vagina

**Published:** 1848

**Authors:** 


Article XII.---*Stricture of the Vagina.*

Dr. C. D. Meigs related the particulars of a case of parturition occurring in a female laboring under stricture of the vagina. On the 11th of April, 1845, the patient was delivered of her second child, and soon after was seized of fever, attended with some peculiar symptoms, which were found to result from a retroversion of the uterus that had occurred before the uterus had become entirely reduced to to its normal size and condition after parturition.---During the remainder of 1845, and the whole of the ensuing year, the patient remained in tolerable health; early in 1847, she began to complain of pelvic pain, attended with some discharge from the vagina. She at first declined submitting to an examination to enable Dr. M. to ascertain the condition of the uterus and vagina.---Her symptoms, however, increasing, she permitted finally the examination to be made: the neck of the uterus was found to be in a condition which induced Dr. M. to suppose, at first, that it was carcinomatous, but on resorting to the speculum, he ascertained that the neck of the uterus was the seat of an extensive ulceration, which did not appear to be of a malignant character. The superficies of the ulcer were equal to that of the fingers of one hand; there was considerable thickening of the parts affected, and portions of the surface were removable in the form of scales. As the ulcer healed, a very considerable contraction of the upper portion of the vagina took place, by which its cavity was reduced in diameter to that of an ordinary silver pencil. By the first of

January of the present year, the lady became pregnant, and about the 10th of July labor pains were experienced. In an examination, to determine the condition of the vagina and os uteri, it was with the greatest dfficulty Dr. M. succeeded in introducing his index finger through the stricture. Opiates, rest, &c., were resorted to in order to keep off the labor. On the 20th of the month, the patient had very severe pains; the membranes, which were uncommonly tough, were gradually protruded beyond the vagina. All the means calculated to produce a relaxation of the vagina were resorted to; sulphate of morphia was administered by the mouth; the patient was immersed in a warm bath; mucilaginous fluids were injected into the vagina; and the strictured portion freely annointed with an ointment containing extract of belladona. The vagina was finally dilated by the advancing head, and on the morning of the 21st the child was born without instrumental assistance.—*Trans. Col. Physi. Phila.*

